# A Combined Impact of Low-Voltage Electrostatic Field and Essential Oil on the Postharvest Properties of Chili Pepper: Insights into Related Molecular Mechanisms

**DOI:** 10.3390/foods13223686

**Published:** 2024-11-19

**Authors:** Xiaoqian Guo, Weihua Liu, Liyong Zhang, Xianghong Wang, Si Mi

**Affiliations:** 1 College of Food Science and Technology, Hebei Agricultural University, No. 2596 Lekai South Road, Baoding 071000, China; xiaoqianguohebau@163.com (X.G.); weihualiuhebau@163.com (W.L.); wangxianghonghebau@163.com (X.W.); 2 Fenghe Agriculture Co., Ltd., Qinhuangdao 066408, China; liyongzhangfenghe@163.com

**Keywords:** chili pepper, postharvest storage, low-voltage electrostatic field, chili pepper leaf essential oil, molecular mechanisms

## Abstract

This research is intended to ascertain the impact of low-voltage electrostatic field (LVEF) together with chili pepper leaf essential oil (CLEO) on the storage quality of chili pepper. Four groups of samples were investigated, namely, control (CK), CLEO, LVEF, and CLEO + LVEF. Chili pepper from the CLEO + LVEF group reduced the weight loss and malondialdehyde content but improved the ascorbic acid contents, antioxidant potential, firmness, and color attributes. CLEO and LVEF could maintain the integral structure and stability of the cell wall by suppressing the activities of hydrolases of pectin, cellulose, and hemicellulose. The positive role of CLEO + LVEF on the color quality was explained by the significantly higher chlorophyll content and lower activities of chlorophyllase, pheophytinase, and Mg-dechelatase compared to the CK group. Taken together, all data provide supporting evidence for a synergistic effect of CLEO and LVEF on the enhancement of postharvest traits of chili peppers.

## 1. Introduction

Chili pepper is one of the most popular vegetable crops worldwide. It is susceptible to quality deterioration like water loss, fruit decay, and yellowing, which could cause great economic losses and shorten the market life of chili peppers [1]. To solve these problems, previous studies have focused on the exploration of novel strategies to alleviate the quality loss and increase the storage lifetime of harvested chili peppers. These methods include gas (e.g., ozone and hexanal vapor) fumigation, intermittent warming, and chemical (e.g., chitosan and chlorine dioxide) coating [2,3]. Our group examined the feasibility of cold-water precooling to improve the physicochemical traits of chili pepper fruit [4].

In recent years, many physical field-assisted techniques have been applied for the preservation of agro-products. Low-voltage electrostatic field (LVEF) with a range of 0~5 kV is a physical alternative to keep the storage features and enhance the market time of livestock and poultry products [5]. Its positive influence on plant-based foodstuffs, such as freshly cut pineapple [6] and button mushrooms [7], was also reported. LVEF is superior to other approaches for its sound efficiency and easy applicability but low energy cost [5]. The underlying mechanisms could be the suppression of microbial and enzyme activities as well as water loss [6,7]. Up to now, little evidence has been available with respect to the role of LVEF on the storage properties of chili peppers.

In addition to all methods mentioned above, surface coating with essential oil isolated from *Heracleum persicum* fruit was also examined for the preservative impact of bell pepper [8]. Research data proved that essential oil was a safe and workable treatment to enhance the marketability of sweet bell pepper [8]. Our group obtained essential oil from chili pepper leaves (CLEO), which had antioxidant and antimicrobial capacities. Thus, we propose that CLEO has the potential to be applied for the improvement of postharvest traits of chili peppers.

The current research was performed to examine the influence of low-voltage electrostatic field and chili pepper leaf essential oil applied in a single or combined way on the preservation of chili pepper fruit as well as to elucidate the underlying molecular mechanisms. A series of quality features, especially firmness and color, would be addressed, and the related molecular mechanisms would be elucidated.

## 2. Materials and Methods

### 2.1. Chemicals and Assay Kits

Analytical-grade anhydrous ethanol was purchased from Huihang Chemical Technology Co., Ltd. (Tianjin, China). Sulfuric acid and acetone of analytical grade were ordered from Kemiou Chemical Reagent Co., Ltd. (Tianjin, China). Phosphate buffer solution (PBS, pH = 7.2~7.4) was ordered from Solarbio Science & Technology Co., Ltd. (Beijing, China).

Commercial test kits applied for the content measurement of protopectin (Product# G0703W), water-soluble pectin (Product# G0704W), chelate-soluble pectin (Product# G0705W), and sodium carbonate-soluble pectin (Product# G0706W) were purchased from Geruisi Biotechnology Co., Ltd. (Suzhou, China). Test kits for the examination of malondialdehyde level (Product# BC0025), cellulose content (Product# BC4285), hemicellulose content (Product# BC4445), cellulase activity (Product# BC2545), peroxidase activity (Product# BC0090), and catalase activity (Product# BC0200) were obtained from Solarbio Science & Technology Co., Ltd. (Beijing, China). Test kits for the analysis of polygalacturonase activity (Product# BS-E1801301), pectin methylesterase activity (Product# HBDY45459), and β-glucosidase activity (Product# BS-E1852001) were provided by Jinyou Technology Co., Ltd. (Beijing, China). Chlorophyllase activity (Product# HB045X-Pt), pheophytinase activity (Product# HB086X-Pt), and Mg-dechelatase activity (Product# HB409X-Pt) were obtained from Hengyuan Biological Co. Ltd. (Beijing, China).

### 2.2. Sample Collection, Treatment and Grouping

Commercial mature chili pepper (*Capsicum annuum* L.) was bought from a local farm (38°52′00.00″ N and 115°29′00.00″ E, altitude 20 m). The fruit chili pepper was harvested and transferred to the laboratory within two hours under 25 °C and 30% humidity. Eighty chili peppers with uniform size, consistent color, and without diseases or visible damage were applied for the following treatment.

These fruits were gently cleaned with a cotton cloth and then randomly divided into four groups: control (CK), chili pepper leaf essential oil (CLEO), low-voltage electrostatic field (LVEF), and chili pepper leaf essential oil combined with low-voltage electrostatic field (CLEO + LVEF) groups. Each treatment was performed on 18 individual chili pepper fruits. Chili pepper leaf essential oil was collected by supercritical CO_2_ extraction (25.0–26.0 MPa, 6 h, 50 ± 2 °C) and coated on the chili pepper surface gently and evenly at 16.6 mg/cm^2^. A low-voltage electrostatic field at 1.6 ± 0.2 kV was created by an instrument from BOMEITE (Binzhou, China) and lasted for the whole storage period. After treatment, all samples were put into a polyethylene bag (30 cm × 40 cm, 0.07 mm in thickness), which had two holes (6 mm i.d.) located in the upper corner. Each pack of chili peppers was held under a temperature of 10 ± 2 °C and a relative humidity of 90–95%. The sampling time was at 0, 7, 14, and 21 days during the storage time.

### 2.3. Determination of Physicochemical Properties of Chili Pepper

A JM-B50002 electronic balance (Sartorius Scientific Instruments Co., Ltd., Beijing, China) was utilized to evaluate the reduction in the weight of chili peppers with reference to the published procedures [4]. The proportion of the weight before storage was recorded as the weight loss (%) of each pack of chili peppers. There were three measurements (n = 3) for each set of samples.

After being cut into 1 cm square pieces from the central part, the fruit of chili pepper was assessed for firmness (N) using a texture analyzer (Food Technology Cooperation, McLean, VA, USA) with reference to the previously published protocols [3,4]. The analyzer was operated by setting the parameters as follows: test speed, 1 mm/s; initial force, 5× *g*; compression degree, 50%; pause time, 2 s. There were three measurements (n = 3) for each set of samples.

The color quality of chili pepper was indicated by L*, a*, and b* values, which were determined with a color meter (WSC-213, Yidian Physical Optics Instrument Co., Ltd., Shanghai, China) with reference to the previously published protocols [3,4]. There were three measurements (n = 9) for each set of samples.

A digital hand-held refractometer (RSD200, AS ONE, Tokyo, Japan) was utilized to evaluate the soluble solid content present in the chili pepper samples with reference to the previously published protocols [3,4]. The lens of the refractometer was calibrated with distilled water and then carefully wiped until dry. Each processed chili pepper sample was evenly mixed, squeezed, filtered with gauze, and then determined. The determined result was presented as a proportion (%) of fresh fruit weight. There were three measurements (n = 3) for each set of samples.

The vitamin C content of the chili pepper was assessed by titration with 2,6-dichloroindophenol with reference to the previously published protocols [3,4]. In brief, around 100 g of sample tissue was homogenized with the same amount of 2% oxalic acid. Followingly, 20 g of the sample mixture was further diluted with 1% oxalic acid to obtain a final volume of 100 mL. The calibrated 2,6-dichloroindophenol solution was used to titrate 10 mL of supernatant until the pink color came out and lasted for 15 s. Distilled water instead of sample supernatant was utilized for the blank control. There were three measurements (n = 3) for each set of samples.

### 2.4. Determination of the Antioxidant Capacities of Chili Pepper

The malondialdehyde (MDA) content of chili pepper was assessed in compliance with the protocols established by the assay kit and with reference to the published method [4]. Briefly, an amount of 0.1 g chili pepper fruit was homogenized with 1 mL of 10% (*w*/*v*) trichloroacetic acid and subjected to centrifugation at 4 °C at 4542× *g* for 10 min to collect the supernatant. Then, 100 μL of supernatant was subjected to 5% (*w*/*v*) thiobarbituric acid (300 μL) and 10% (*w*/*v*) trichloroacetic acid (100 μL), kept in boiling water for 1 h, and centrifuged at 7097× *g* for 10 min after cooling to ambient temperature. Absorbance was recorded at wavelengths of 532 and 600 nm using a UV–Visible Spectrophotometer (UV-2800A, Uniko Co., Ltd., Shanghai, China). There were three measurements (n = 3) for each set of samples.

Peroxidase (POD) activity of the chili pepper was assessed in compliance with the protocols developed by the commercial assay kit and by the published paper [9]. Around 0.1 g chili pepper fruit was homogenized with 1 mL of extraction solution on ice and exposed to 10 min centrifugation at 8000× *g* and 4 °C to collect the supernatant. A mixed solution containing 20 mM guaiacol (520 μL), 50 mM phosphate buffer (pH = 6.8, 130 μL), 0.3% (*w*/*v*) H_2_O_2_ (135 μL), and distilled water (270 μL) was stored at 37 °C for 10 min and then reacted with 15 μL of supernatant. The absorbance at the beginning and 1 min after reaction was measured at a wavelength of 470 nm using a UV–Visible Spectrophotometer (UV-2800A, Uniko Co., Ltd., Shanghai, China). POD activity was calculated as the change rate of the absorbance value. There were three measurements (n = 3) for each set of samples.

Catalase (CAT) activity of the chili pepper was assessed in reference to the protocols provided by the commercial assay kit and to the published method [9]. Chili pepper fruit (0.1 g) was ground with 1 mL extraction solution on ice and then exposed to 10 min centrifugation at 8000× *g* and 4 °C to obtain the supernatant. Subsequently, a reaction solution comprising 50 mM phosphate buffer (pH = 7) and 15 mM H_2_O_2_ was combined with 35 μL of supernatant. The absorbance at the beginning and after 1 min of the reaction was evaluated at a wavelength of 240 nm using a UV–Visible Spectrophotometer (UV-2800A, Uniko Co., Ltd., Shanghai, China). CAT activity was calculated as the change rate of the absorbance value. There were three measurements (n = 3) for each set of samples.

### 2.5. Quantification of Cell Wall Polysaccharides in the Chili Pepper Fruit

As directed by the assay kit instructions, the protopectin and water-soluble pectin (WSP) contents of the chili pepper were ascertained. Chili pepper powder (0.01 g) was dissolved in 80% (*v*/*v*) ethanol (1.5 mL), stored at 85 °C for 10 min, and then centrifuged at 4600× *g* for 10 min to remove the interfering substances. The centrifugation process was conducted twice, and the pellets were combined. After that, the pellet was centrifuged for 10 min at 4600× *g* after being incubated for 30 min at 50 °C with 1 mL of distilled water to dissolve the pectin fraction. The supernatant was collected into an eppendorf tube to determine the level of soluble pectin, whereas the pellet was utilized to determine the protopectin content. A wavelength of 530 nm was used to measure the absorbance using a UV–Visible Spectrophotometer (UV-2800A, Uniko Co., Ltd., Shanghai, China). There were three measurements (n = 3) for each set of samples.

The contents of chelate-soluble pectin (CSP) and sodium carbonate-soluble pectin (SSP) in the chili pepper were determined in compliance with the instructions of the test kits. Chili pepper powder (0.02 g) was added to 80% (*v*/*v*) ethanol (1.5 mL), retained for 10 min in an 85 °C water bath, and then centrifuged for 10 min at 4600× *g* to eliminate sugar and other interfering materials. The centrifugation was repeated twice, and the combined pellet was mixed with 1 mL of extract A. Then, the resulting sample was put into a 95 °C water bath for 15 min and exposed to 10 min centrifugation at 4600× *g* to collect the pellet, which was decolorized with acetone. After dryness, the pellet was added to 1 mL of extract B, shaken for 1 h, and centrifuged at 4600× *g* for 10 min. The supernatant was obtained to measure the CSP and SSP levels. Absorbance was measured at a wavelength of 530 nm using a UV–Visible Spectrophotometer (UV-2800A, Uniko Co., Ltd., Shanghai, China). There were three measurements (n = 3) for each set of samples.

The cellulose content of chili pepper was assessed with a commercially available test kit. Around 0.3 g of chili pepper sample was weighed, combined thoroughly with 1 mL of 80% (*v*/*v*) ethanol, and then incubated for 20 min in a 90 °C water bath. Once the mixture had reached the ambient temperature, it was centrifuged for 10 min at 6000× *g* to collect the pellet. The pellet was then washed twice sequentially with 80% (*v*/*v*) ethanol and acetone solution to obtain the crude extract of the cell wall. Followingly, 1 mL of 90% (*w*/*v*) dimethyl sulfoxide solution was subjected to the crude extract, soaked for 15 h to remove starch, and then exposed to 10 min centrifugation at 6000× *g* to obtain a pellet. The pellet was cleaned twice with distilled water and then dried at 60 °C for 12 h in order to extract the cell wall components. Around 5 mg of dried cell wall materials was mixed with 500 μL of distilled water for homogenization, followed by a slow addition of 0.75 mL of concentrated sulfuric acid. The resulting mixture was gently stirred for 30 min in an ice bath and exposed to 10 min centrifugation at 8000× *g* at 4 °C. The supernatant was diluted 20 times with distilled water. A volume of 150 μL diluted solution was subjected to 35 μL of 2% (*w*/*v*) anthrone and 315 μL of concentrated sulfuric acid. After 10 min at 95 °C, the absorbance was measured at 620 nm using a UV–Visible Spectrophotometer (UV-2800A, Uniko Co., Ltd., Shanghai, China). There were three measurements (n = 3) for each set of samples.

The hemicellulose content of chili pepper was evaluated in compliance with the instructions provided by the assay kit. Around 0.05 g of chili pepper powder was weighed and reacted with 1 mL of 80% (*v*/*v*) ethanol at 90 °C for 10 min. The solution was allowed to cool to room temperature before being centrifuged at 8000× *g* for 10 min. The pellet was then collected and dried to a constant weight after being washed three times with 1 mL of distilled water. The pellet was added to 500 μL of extract I and reacted in a 90 °C water bath for 1 h. Following a cooling period to room temperature, 500 μL of extract II was added, followed by 10 min centrifugation at 8000× *g*. A volume of 80 μL supernatant, 80 μL 3,5-dinitrosalicylic acid, and 160 μL distilled water was added sequentially, mixed well, and then subjected to 10 min centrifugation at 8000× *g*. A wavelength of 540 nm was applied to evaluate the absorbance using a UV–Visible Spectrophotometer (UV-2800A, Uniko Co., Ltd., Shanghai, China). There were three measurements (n = 3) for each set of samples.

### 2.6. Determination of the Enzyme Activities Related to Polysaccharide Metabolism

Polygalacturonase (pectin methylesterase or β-glucosidase) activity of the chili pepper was ascertained in compliance with the assay kit’s instructions. To obtain the supernatant, approximately 0.1 g of chili pepper tissue was homogenized using 0.9 mL PBS buffer and centrifuged at 640× *g* for 20 min. Then, 10 μL of supernatant and 40 μL of sample diluent were sequentially added to a micropore coated with polygalacturonase (pectin methylesterase or β-glucosidase) antibody, followed by the supplement of 100 μL of horseradish peroxidase (HRP) labeled detection antibody. The microtiter plate was sealed with a film and kept at 37 °C for one hour. After washing thoroughly five times, the tetramethylbenzidine substrate was utilized to produce color. A wavelength of 450 nm was applied to evaluate the absorbance using a UV–Visible Spectrophotometer (UV-2800A, Uniko Co., Ltd., Shanghai, China). There were three measurements (n = 3) for each set of samples.

The cellulase activity of the chili pepper was ascertained in compliance with the assay kit’s instructions. After homogenizing approximately 0.1 g of chili pepper tissue in an ice bath with 1 mL extract, the mixture was centrifuged at 8000× *g* for 10 min at 4 °C in order to collect the supernatant. Followingly, 50 μL of enzyme solution, 200 μL of 0.5% (*w*/*v*) carboxymethyl cellulose sodium solution, 50 μL of distilled water, and 50 μL of supernatant were added to a centrifuge tube sequentially, mixed well, kept in a 40 °C water bath for 30 min, and then immediately transferred to boiling water for 15 min to obtain the saccharified solution. After these, saccharified liquid (15 μL) was mixed well with 3,5-dinitrosalicylic acid (35 μL) in a boiling water bath for 15 min to develop color. When cooled down, 250 μL of distilled water was combined and ready for absorbance measurement at a wavelength of 540 nm using a UV–Visible Spectrophotometer (UV-2800A, Uniko Co., Ltd., Shanghai, China). There were three measurements (n = 3) for each set of samples.

### 2.7. Quantification of Chlorophyll Constitutes in the Postharvest Chili Pepper

Chlorophyll content was ascertained in compliance with the previously published method [10] with some adjustments. Approximately 0.2 g of chili pepper tissue was weighed, added to 10 mL of 95% (*v*/*v*) ethanol, mixed well, and maintained in the dark for 12 h. Subsequently, the mixture was subjected to a 10 min centrifugation at 8000× *g* to extract the supernatant. The absorbance was recorded at 470, 649, and 665 nm, with 95% (*v*/*v*) ethanol as the blank control. There were three measurements (n = 3) for each set of samples. The calculation formulas were as follows:

Chlorophyll a (mg/g) = (13.95 × A_665_ − 6.88 × A_649_) × V/1000 m

Chlorophyll b (mg/g) = (24.96 × A_649_ − 7.32 × A_665_) × V/1000 m

Chlorophyll (mg/g) = (ρ_a_ + ρ_b_) × V/1000 m

where ρ_a_ is the mass concentration of chlorophyll a (mg/mL), ρ_b_ is the mass concentration of chlorophyll b (mg/mL), V represents the total volume of sample extract (mL), and m represents sample weight (g).

### 2.8. Determination of the Enzyme Activities Related to Chlorophyll Metabolism

The chlorophyllase (pheophytinase or Mg-dechelatase) activity of the chili pepper was ascertained in compliance with the assay kit’s instructions. The chili pepper tissue (0.1 g) was ground with 0.9 mL PBS buffer and exposed to a 20 min centrifugation at 640× *g* to obtain the supernatant. The microtiter plate was coated with purified plant chlorophyllase (plant pheophytinase or plant Mg-dechelatase) antibody and filled with 40 μL sample diluent and 10 μL supernatant for each well. After that, the plate was covered with parafilm and allowed to stay at 37 °C for half an hour. A tetramethylbenzidine substrate was added to each well to create color after a thorough washing. A wavelength of 450 nm was used to determine the absorbance. There were three measurements (n = 3) for each set of samples.

### 2.9. Data Processing and Statistics

The mean value and standard deviation (SD) of each result were displayed. Notable variations were denoted as * *p* < 0.05, ** *p* < 0.01, *** *p* < 0.001 and **** *p* < 0.0001 (CELO vs. CK), # *p* < 0.05, ## *p* < 0.01, #### *p* < 0.001 and #### *p* < 0.0001 (LVEF vs. CK), ▽ *p* < 0.05, ▽▽ *p* < 0.01, ▽▽▽ *p* < 0.001, and ▽▽▽▽ *p* < 0.0001 (CLEO + LVEF vs. CK). Data were processed using Excel v2021 software. The SPSS 23.0 software was applied to conduct the Pearson correlation analysis. Figures were prepared using GraphPad Prism 8.0 software (San Diego, CA, USA).

## 3. Results and Discussion

### 3.1. Effect of Different Treatments on the Physicochemical Properties of Postharvest Chili Pepper

**Figure 1:**
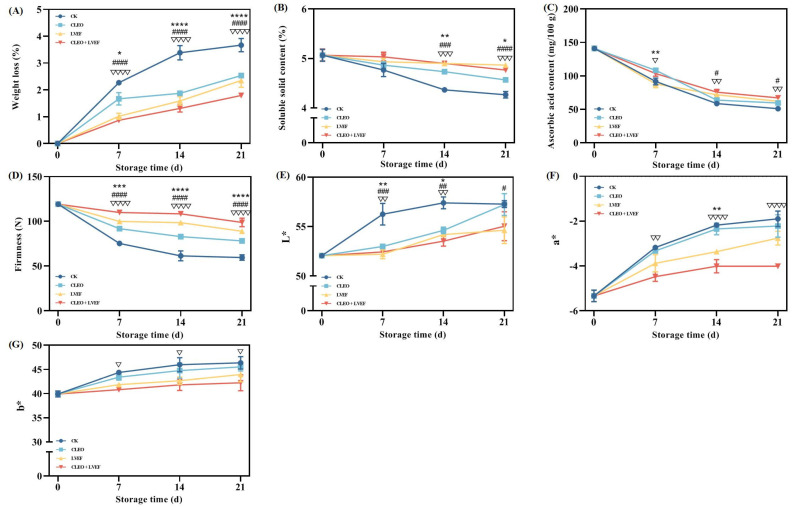
Effect of different treatments on the (**A**) weight loss, (**B**) soluble solid content, (**C**) ascorbic acid content, (**D**) firmness, (**E**) L* value, (**F**) a* value, and (**G**) b* value of chili pepper over a 0–21 d storage period. CK, control group; CLEO, chili pepper leaf essential oil; LVEF, low-voltage electrostatic field; CLEO + LVEF, chili pepper leaf essential oil together with low-voltage electrostatic field. * *p* < 0.05, ** *p* < 0.01, *** *p* < 0.001, **** *p* < 0.0001, CLEO compared to control (CK); # *p* < 0.05, ## *p* < 0.01, ### *p* < 0.001, #### *p* < 0.0001, LVEF compared to control (CK); ▽ *p* < 0.05, ▽▽ *p* < 0.01, ▽▽▽ *p* < 0.001, ▽▽▽▽ *p* < 0.0001, CLEO + LVEF compared to control (CK).

An enhanced positive effect was achieved when LVEF and CLEO were applied together. All chili pepper samples showed an upward trend in weight loss (Figure 1A). Those under no treatment (CK group) exhibited the most serious weight loss during the 21-day storage period. In contrast, the smallest weight loss was shown in the samples from the LVEF + CLEO group (Figure 1A). Weight loss is a vital feature to evaluate the level of freshness and nutritional status of vegetables and fruit [9,11]. The obtained data demonstrate that both chili pepper leaf essential oil and low-voltage electrostatic field were workable to alleviate the reduction in weight of postharvest chili pepper.

Soluble solid content mainly refers to the composition of sugars and organic acids [4]. A general decline was observed from 5.07% at 0 days to 4.87% (LVEF group), 4.77% (LVEF + CLEO group), 4.57% (CLEO group), and 4.27% (CK group) when the storage was completed (Figure 1B). LVEF appears to be more positive than CLEO treatment in terms of maintaining the soluble solid content of chili pepper. This could be plausibly due to the inhibition of LVEF on the metabolic enzymes, thereby delaying the loss of soluble solid content [6,12].

A steady decreasing trend from 141.05 mg/100 g at 0 days to 67.21 mg/100 g (LVEF + CLEO group), 62.01 mg/100 g (LVEF group), 59.64 mg/100 g (CLEO group), and 51.12 mg/100 g (CK group) after 21 days of storage (Figure 1C) was achieved in the ascorbic acid content of chili pepper fruit. During the last week (14 days to 21 days) of storage, the ascorbic acid contents determined in the LVEF + CLEO and LVEF groups were remarkably (*p* < 0.05) greater than that of the chili pepper with no treatment (Figure 1C). Ascorbic acid is a dominant component with antioxidant activity in the fruit of chili pepper [4]. The oxidation and breakdown of ascorbic acid occur during the maturation and storage process of vegetables and fruit [6,9,13]. Cheng et al. found that a low-voltage electrostatic field was able to prevent the degradation of ascorbic acid in fresh-cut pineapples [6]. Our research not only confirmed the previous finding but also revealed a combined effect of LVEF and CLEO.

Firmness is an important feature in determining the commercial value of fresh vegetables and fruit [14,15]. According to Figure 1D, the firmness value fell from 119.17 N at the beginning to 59.58 N (CK group), 78.2 N (CLEO group), 88.9 N (LVEF group), and 98.78 N (CLEO + LVEF group) when the storage ended. This matched the findings of our earlier study, which also reported the loss in the texture property in the storage period of chili peppers [3]. Notably, CLEO and LVEF exhibited excellent suppression of the decline of chili pepper firmness, especially a combination of CLEO and LVEF. In addition, Pearson correlation analysis showed a strongly converse link (r = −0.91, *p* < 0.0001) between the firmness and weight loss of chili peppers.

A crucial quality factor influencing the marketability and acceptability of chili peppers among consumers is color [4]. The L* value of all chili peppers showed an upward trend from 52.06 at the beginning to 55.04 (CLEO + LVEF group), 54.63 (LVEF group), 57.24 (CLEO group), and 57.28 (CK group) when the storage was completed (Figure 1E). Similar trends were achieved in the a* (Figure 1F) and b* (Figure 1G) values for all groups of chili pepper. Moreover, the lowest a* (Figure 1F) and b* (Figure 1G) values were always achieved in the chili pepper under a combined treatment of CLEO and LVEF throughout the whole storage period. These data demonstrate that CLEO, together with LVEF, had the most obvious effect in maintaining the green color of fresh chili peppers.

### 3.2. Effect of Different Treatments on the Antioxidant Capacities of Postharvest Chili Pepper

**Figure 2:**
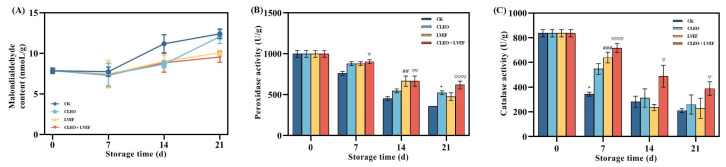
Effect of different treatments on the (**A**) malondialdehyde content, (**B**) peroxidase activity, and (**C**) catalase activity of chili pepper over a 0–21 d storage period. * *p* < 0.05, CLEO compared to control (CK); ## *p* < 0.01, ### *p* < 0.001, LVEF compared to control (CK); ▽ *p* < 0.05, ▽▽ *p* < 0.01, ▽▽▽▽ *p* < 0.0001, CLEO + LVEF compared to control (CK).

Malondialdehyde (MDA) is generated via membrane lipid peroxidation, and its content is positively correlated to the oxidative degree of vegetables and fruit [16]. An increasing trend from 7.85 nmol/g at 0 days to 9.54 nmol/g (LVEF + CLEO group), 10.07 nmol/g (LVEF group), 12.06 nmol/g (CLEO group), and 12.41 nmol/g (CK group) after 21 days of storage was achieved in the MDA level of chili pepper fruit (Figure 2A). These data suggest that LVEF, together with CLEO, exerted a synergistic antioxidant effect by maintaining the lowest MDA content in the chili pepper fruit.

Peroxidase (POD) works to protect vegetables and fruit from oxidative damage by eliminating H_2_O_2_ [17]. All chili pepper samples showed a declining tendency in the POD activity, indicating the loss of antioxidant potentials (Figure 2B). However, both LVEF and CLEO had relatively higher POD activity than that of the CK group during the whole storage cycle (Figure 2B). The highest value of POD activity was constantly present in the LVEF + CLEO group.

Catalase (CAT) exerts an antioxidant effect via scavenging ROS radicals in the plant tissues [18]. Chili peppers from the CK group experienced a sharp loss of CAT activity from 836.20 U/g at 0 days to 343.52 U/g on the 7th day of storage (Figure 2C). LVEF + CLEO group, by contrast, exhibited a slight drop from 836.20 U/g at 0 days to 716.42 U/g on the 7th day of storage (Figure 2C). Furthermore, the LVEF + CLEO group had the highest CAT activity among others throughout the storage life.

All the above data imply that both low-voltage electrostatic field and chili pepper leaf essential oil were beneficial in reducing the activity loss of antioxidant enzymes and further suppressing the MDA levels in the chili pepper fruit. This was partially consistent with the previous reports, which attributed the antioxidant ability of LVEF to the produced electromagnetic radiation [12]. Essential oils isolated from various natural plant resources have also been proven to have antioxidant effects [19,20]. The current study revealed a synergistic role of LVEF and essential oil treatments in maintaining the antioxidant potential of stored chili peppers.

### 3.3. Effect of Different Treatments on the Cell Wall Polysaccharides Composition of Stored Chili Pepper

#### 3.3.1. Protopectin, Water-Soluble Pectin, Chelate-Soluble Pectin Content and Sodium Carbonate-Soluble Pectin Contents

**Figure 3:**
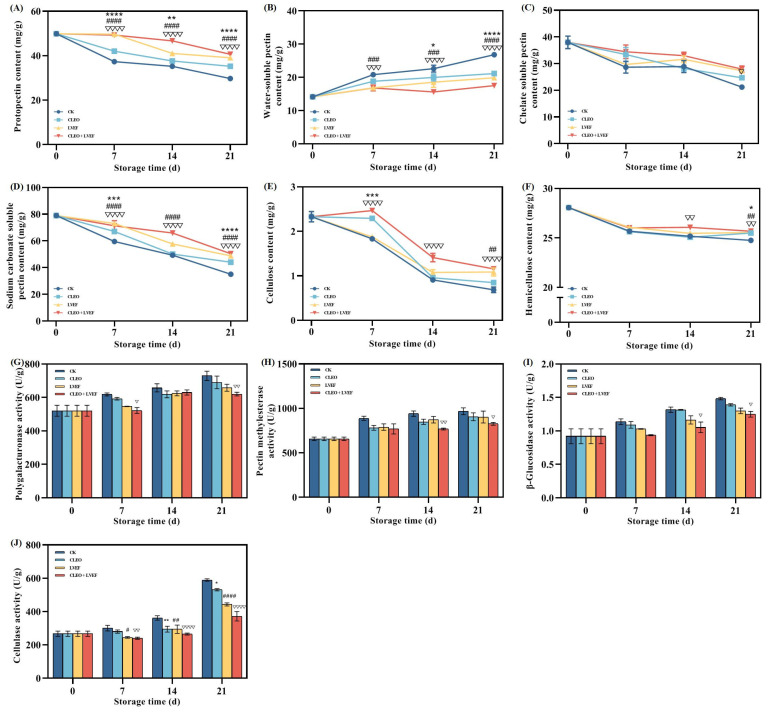
Effect of different treatments on the (**A**) protopectin content, (**B**) water-soluble pectin content, (**C**) chelate soluble pectin content, (**D**) sodium carbonate soluble pectin content, (**E**) cellulose content, (**F**) hemicellulose content, (**G**) polygalacturonase activity, (**H**) pectin methylesterase activity, (**I**) β-glucosidase activity, and (**J**) cellulase activity of chili pepper over a 0–21 d storage period. * *p* < 0.05, ** *p* < 0.01, *** *p* < 0.001, **** *p* < 0.0001, CLEO compared to control (CK); # *p* < 0.05, ## *p* < 0.01, ### *p* < 0.001, #### *p* < 0.0001, LVEF compared to control (CK); ▽ *p* < 0.05, ▽▽ *p* < 0.01, ▽▽▽ *p* < 0.001, ▽▽▽▽ *p* < 0.0001, CLEO + LVEF compared to control (CK).

Pectin is a family member of polysaccharides closely related to the structural rigidity of plant cell walls [21]. Figure 3A illustrates the level of protopectin determined in the chili pepper samples. All chili peppers experienced a descending trend in the protopectin content from 49.87 mg/g at 0 days to 29.74 mg/g (CK group), 35.23 mg/g (CLEO group), 39.10 mg/g (LVEF group), and 40.61 mg/g (CLEO + LVEF group) till the end of storage (Figure 3A). Decomposition of protopectin occurs during fruit ripening, which is accompanied by tissue softening [22]. Correlation analysis detected a significantly negative link (r = 0.89, *p* < 0.0001) between protopectin level and firm properties of chili pepper (Table 1). All treatments alleviated the loss of protopectin within the entire storage period compared to the CK group (Figure 3A). The CLEO + LVEF group maintained the highest content of protopectin, among others.

The separation of protopectin from cellulose leads to the production of WSP fractions [23]. This can be verified by a converse correlation between the protopectin and WSP concentrations (r = −0.88, *p* < 0.0001). Figure 3B demonstrates a gradual lift of WSP content from 14.13 mg/g when the storage was started to 17.52 mg/g (CLEO + LVEF group), 19.90 mg/g (LVEF group), 21.16 mg/g (CLEO group), and 26.80 mg/g (CK group) when the storage was completed. The increase in WSP level could lead to the disappearance of intercellular adhesion and further aggravate the plant cell wall depolymerization [12]. The WSP content and firmness had a strongly reverse relationship (r = −0.88, *p* < 0.0001) (Table 1).

Chelate-soluble pectin (CSP) with low water solubility is ionically bound pectin by calcium bridges [24]. CSP content showed a downward trend in all groups of chili peppers (Figure 3C). When the storage was completed, the CSP level determined in the CK group (21.20 mg/g) was obviously less than that in the CLEO + LVEF group (*p* < 0.05). There was a negative link present between the CSP content and firmness value of chili pepper (r = 0.68, *p* < 0.0001). In addition to CSP, sodium carbonate-soluble pectin (SSP) is another type of water-insoluble pectin combined with other cell wall components via covalent bonds [24]. Tao et al. revealed that a reduction in SSP caused the breakdown of cell walls and thus led to fruit softening [25]. This was confirmed by the Pearson correlation data (r = −0.78, *p* < 0.0001) between the SSP concentration and the texture property of chili pepper (Table 1). In the present study, the SSP content declined steadily during the storage period (Figure 3D). The CK group consistently had the lowest value. There have been previous studies revealing that CSP and SSP fractions prevented the depolymerization and dissolution of cell wall polysaccharides [25,26]. The obtained data suggest that CLEO + LVEF postponed the softening and preserved the rigidity of the chili pepper cell wall by keeping the proportions of water-insoluble pectin at higher levels.

#### 3.3.2. Cellulose and Hemicellulose Contents

Cellulose is another dominant polysaccharide component mainly present in the primary and secondary plant cell walls [27,28,29]. The cellulose content of all chili pepper samples demonstrated a declining trend for the duration of storage (Figure 3E). When the storage stopped, the cellulose contents of the CLEO, LVEF, and CLEO + LVEF groups were 1.23, 1.58, and 1.69 times greater than the data of the CK group (Figure 3E), suggesting all treatments were effective in lessening the degradation of cellulose. Furthermore, a strongly positive connection (r = 0.64, *p* < 0.0001) was observed between the cellulose concentration and the firmness of chili pepper (Table 1).

Hemicellulose works to maintain the plant cell morphology via connection with microfibrils [15,27]. Similar to cellulose, hemicellulose content also showed a decreasing tendency as storage extended (Figure 3F). A significant difference (*p* < 0.01) was achieved in the hemicellulose content between the CLEO + LVEF group (25.68 mg/g) and the CK group (24.76 mg/g). Furthermore, a strongly positive connection (r = 0.69, *p* < 0.0001) was observed between the hemicellulose level and the firmness of chili pepper (Table 1). Fruit softening is usually accompanied by the breakdown of cellulose and hemicellulose, resulting in damage to the cell wall network [26]. Taken together, the positive effect of CLEO + LVEF on firmness could be plausibly explained by inhibiting the breakdown of cellulose and hemicellulose.

#### 3.3.3. Polygalacturonase, Pectin Methylesterase, β-Glucosidase, and Cellulase Activities

A variety of enzymes are involved in the metabolism of cell wall polysaccharides, including cellulose, hemicellulose, and pectin [6,14].

Polygalacturonidase (PG) works for pectin disassembly by targeting α-1,4-linked D-galacturonic acid [14]. As demonstrated in Figure 3G, an overall upward tendency was noted in the PG activity for each group of chili peppers. This suggests the exacerbation of pectin hydrolysis throughout the storage lifespan. The most obvious variation occurred in the CK group from 520.40 U/g at the beginning to 729.43 U/g when the storage ended (Figure 3G). This agrees with the change in pectin fractions discussed above, which can be verified by the correlation data (Table 2).

Pectin de-esterification is primarily caused by pectin methylesterase (PME), which eliminates the methyl group from galacturonic acid [23,29]. A gradual lifting was shown in the PME activity for all groups of chili peppers (Figure 3H). When the storage finished, PME activities were 967.34, 906.02, 902.64, and 826.30 U/g for CK, CLEO, LVEF, and CLEO + LVEF groups, respectively (Figure 3H). This indicates that CLEO + LVEF inhibited the PME activity, thus delaying the decomposition of pectin molecules. Except for WSP, PME activity had significantly negative correlations with all pectin, cellulose, and hemicellulose components (Table 3).

β-Glucosidase (β-Glu), a member of cellulase, acts to produce glucose from cell wall polysaccharides via breaking the β-D-glycosidic bond [29,30]. Like PG and PME, an upward trend was also shown in the β-Glu activity within the duration of storage (Figure 3I). The highest activity was always present in the CK group, while the lowest value was observed in the CLEO + LVEF group (Figure 3I). β-Glucosidase activity had significantly negative associations with PP (r = −0.85 and *p* < 0.0001), CSP (r = −0.78 and *p* < 0.0001), SSP (r = −0.89 and *p* < 0.0001), cellulose (r = −0.84 and *p* < 0.0001), and hemicellulose (r = −0.69 and *p* = 0.004) contents, as shown in Table 4. These results demonstrate that CLEO + LVEF was effective in reducing the β-Glu activity and further preserved the structural integrity of the cell wall. A reverse relationship between the firmness and β-Glu activity of the chili pepper was found using Pearson correlation analysis (Table 1).

Cellulase (Cx) works to accelerate the hydrolysis of cellulose via targeting acting on the β-1,4-glucoside bond [14,18]. As shown in Figure 3J, an overall rise was shown in the cellulase activity for all groups of chili peppers, implying the deterioration of cellulose components. When storage was completed, the Cx activity of the CK group reached 589.07 U/g, being 1.11, 1.33, and 1.78 times greater than the values of CLEO, LVEF, and CLEO + LVEF groups, respectively (Figure 3J). Pearson correlation analysis showed negative relations of Cx activity with firmness (r = −0.65, *p* < 0.0001), cellulose content (r = −0.69, *p* < 0.0001), and hemicellulose content (r = −0.45, *p* = 0.004) (Table 1 and Table 5).

All the above data provide evidence to clarify the molecular mechanism behind the benefits of LVEF and CLEO for preserving the firm feature of stored chili peppers. LVEF, together with CLEO, suppressed the enzyme activity, which catalyzed the hydrolysis of cell wall polysaccharides. The rigidity and stability of the cell wall inhibited the softening and decay of postharvest chili pepper.

### 3.4. Effect of Different Treatments on the Chlorophyll Degradation of Stored Chili Pepper

#### 3.4.1. Content of Chlorophyll Fractions

**Figure 4:**
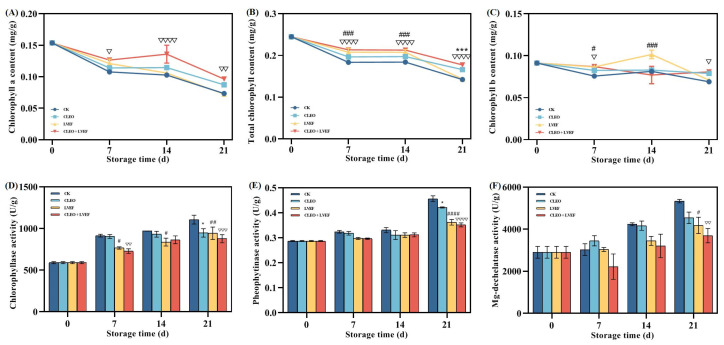
Effect of different treatments on the (**A**) chlorophyll a content, (**B**) chlorophyll b content, (**C**) total chlorophyll content, (**D**) chlorophyllase activity, (**E**) pheophytinase activity, and (**F**) Mg-dechelatase activity of chili pepper over a 0–21 d storage period. * *p* < 0.05, *** *p* < 0.001, CLEO compared to control (CK); # *p* < 0.05, ## *p* < 0.01, ### *p* < 0.001, #### *p* < 0.0001, LVEF compared to control (CK); ▽ *p* < 0.05, ▽▽ *p* < 0.01, ▽▽▽ *p* < 0.001, ▽▽▽▽ *p* < 0.0001, CLEO + LVEF compared to control (CK).

Chlorophyll contributes to the green color of chili peppers. Its degradation is a major cause of the yellowing and senescence of chili pepper [31]. As illustrated in Figure 4A–C, the contents of chlorophyll a, b, and total chlorophyll showed a declining pattern for the duration of the storage time, suggesting the deterioration of the green color of chili pepper. This could be verified by the significantly negative correlations between total chlorophyll content and a* (r = −0.69, *p* < 0.0001) as well as b* value (r = −0.63, *p* < 0.0001). Statistical analysis found that the levels of chlorophyll a (0.10 mg/g), b (0.08 mg/g), and total chlorophyll (0.18 mg/g) in the CLEO + LVEF group were notably *(p* < 0.05) greater than those of the CK group (Figure 4A–C).

#### 3.4.2. Chlorophyllase, Pheophytinase, and Mg-Dechelatase

Chlorophyllase catalyzes the conversion of chlorophyll a (blue–green) to chlorophyll a (green) [32]. An overall climbing trend was observed in the chlorophyllase activity from 590.50 U/g at 0 days to 1107.47 U/g (CK group), 947.64 U/g (CLEO group), 943.61 U/g (LVEF group), and 878.56 U/g (CLEO + LVEF group) when the storage finished (Figure 4D). This suggests an accelerated deterioration of chlorophyll a, which could be confirmed by the negative association between chlorophyll a content and chlorophyllase activity (r = −0.74, *p* < 0.0001), shown in Table 6.

Pheophytinase regulates the conversion of pheophytin a (olive–brown) to pheophorbide a (brown), a key step affecting the yellowing and senescence of vegetables [32]. Pheophytinase activity determined in the CK and CLEO groups changed greatly from 0.29 U/g at 0 days to 0.46 U/g and 0.42 U/g, respectively, when the storage finished (Figure 4E). Pheophytinase activity and b* value had a positive correlation according to Pearson correlation analysis (r = 0.59, *p* < 0.0001) while conversely associated with total chlorophyll content (r = −0.80, *p* < 0.0001) (Table 7). The lowest activity of pheophytinase was always detected in the chili pepper from the CLEO + LVEF group (Figure 4E), demonstrating the maintenance of green color properties.

Mg-dechelatase is a key enzyme for the production of pheophorbide a by removing mg^2+^ from chlorophyllide, resulting in the occurrence of brown color [33]. Like chlorophyllase and pheophytinase, a general rise was shown in the Mg-dechelatase activity for all groups of chili peppers (Figure 4F), implying the loss of green color. When the storage was completed, the Mg-dechelatase activity of the CK group was 1.17, 1.27, and 1.44 times greater than that of the CLEO, LVEF, and CLEO + LVEF groups, respectively (Figure 4F). The Mg-dechelatase activity was also conversely related to the total chlorophyll level (r = −0.65, *p* < 0.0001) but positively related to the b* value (r = 0.48, *p* = 0.002), indicated in Table 8.

The improving impact of LVEF and CLEO on the color attribute of chili pepper was explained by the chlorophyll metabolism and involved enzyme activities. Both LVEF and CLEO had inhibiting effects on the enzymes responsible for weakening the green color of chili peppers.

## 4. Conclusions

The present research looked into how LVEF and CLEO worked on the physicochemical characteristics of chili peppers during a 0–21 storage period. Both LVEF and CLEO prove to be feasible to mitigate the quality deterioration of stored chili peppers. The optimal results were achieved in the chili pepper under a combined treatment of LVEF and CLEO, which had a 2.04, 1.26, and 2.11 times reduction in weight loss, malondialdehyde content, and a* value, but 1.66, 1.12, and 1.31 times lifting in firmness, soluble solids, and ascorbic acid contents compared to the non-treated group. LVEF and CLEO significantly hindered the decomposition of pectin, cellulose, and hemicellulose from keeping the intact structure of the cell wall and further alleviated the softening of chili pepper. The positive impact on the color was attributed to the prevention of chlorophyll degradation via suppressing the activities of chlorophyllase, pheophytinase, and Mg-dechelatase. LVEF, together with CLEO, proves to be an alternative strategy to improve the storage properties of chili peppers. Future work could be performed to evaluate the expression levels of key genes responsible for the regulation of the physicochemical qualities of chili peppers.

## Figures and Tables

**Table 1:** Pearson correlation matrix of firmness, cell wall polysaccharides, and related hydrolase activity in the chili pepper fruit during a 0–21 d storage period.

	Firmness vs. PP Content	Firmness vs. WSP Content	Firmness vs. CSP Content	Firmness vs. SSP Content	Firmness vs. CL Content	Firmness vs. HCL Content	Firmness vs. PG Activity	Firmness vs. PME Activity	Firmness vs. β-Glu Activity	Firmness vs. Cx Activity
**Pearson r**	0.89	−0.89	0.68	0.78	0.64	0.69	−0.71	−0.75	−0.76	−0.65
***p*-value**	<0.0001	<0.0001	<0.0001	<0.0001	<0.0001	<0.0001	<0.0001	<0.0001	<0.0001	<0.0001

Note: PP, protopectin; WSP, water-soluble pectin; CSP, chelate-soluble pectin; SSP, sodium carbonate-soluble pectin; CL, cellulose; HCL, hemicellulose; PG, polygalacturonase; PME, pectin methylesterase; β-Glu, β-glucosidase; Cx, cellulase.

**Table 2:** Pearson correlation matrix of polygalacturonase activity and cell wall polysaccharides in the chili pepper fruit during a 0–21 d storage period.

	PG Activity vs. PP Content	PG Activity vs. WSP Content	PG Activity vs. CSP Content	PG Activity vs. SSP Content	PG Activity vs. CL Content	PG Activity vs. HCL Content
**Pearson r**	−0.78	0.68	−0.73	−0.82	−0.80	−0.61
***p*-value**	<0.0001	<0.0001	<0.0001	<0.0001	<0.0001	<0.0001

**Table 3:** Pearson correlation matrix of pectin methylesterase activity and cell wall polysaccharides in the chili pepper fruit during a 0–21 d storage period.

	PME Activity vs. PP Content	PME Activity vs. WSP Content	PME Activity vs. CSP Content	PME Activity vs. SSP Content	PME Activity vs. CL Content	PME Activity vs. HCL Content
**Pearson r**	−0.72	0.75	−0.68	−0.74	−0.68	−0.69
***p*-value**	<0.0001	<0.0001	<0.0001	<0.0001	<0.0001	0.004

**Table 4:** Pearson correlation matrix of β-glucosidase activity and cell wall polysaccharides in the chili pepper fruit during a 0–21 d storage period.

	β-Glu Activity vs. PP Content	β-Glu Activity vs. WSP Content	β-Glu Activity vs. CSP Content	β-Glu Activity vs. SSP Content	β-Glu Activity vs. CL Content	β-Glu Activity vs. HCL Content
**Pearson r**	−0.85	0.78	−0.78	−0.89	−0.84	−0.69
***p*-value**	<0.0001	<0.0001	<0.0001	<0.0001	<0.0001	0.004

**Table 5:** Pearson correlation matrix of cellulase activity and cell wall polysaccharides in the chili pepper fruit during a 0–21 d storage period.

	Cx Activity vs. PP Content	Cx Activity vs. WSP Content	Cx Activity vs. CSP Content	Cx Activity vs. SSP Content	Cx Activity vs. CL Content	Cx Activity vs. HCL Content
**Pearson r**	−0.77	0.73	−0.76	−0.83	−0.69	−0.45
***p*-value**	<0.0001	<0.0001	<0.0001	<0.0001	<0.0001	0.004

**Table 6:** Pearson correlation matrix of chlorophyllase activity and color as well as chlorophyll components in the chili pepper fruit during a 0–21 d storage period.

	CLH Activity vs. L* Value	CLH Activity vs. a* Value	CLH Activity vs. b* Value	CLH Activity vs. Chlorophyll a Content	CLH Activity vs. Chlorophyll b Content	CLH Activity vs. Total Chlorophyll Content
**Pearson r**	0.74	0.71	0.68	−0.74	−0.51	−0.78
***p*** **-value**	<0.0001	<0.0001	<0.0001	<0.0001	0.001	<0.0001

Note: CLH, Chlorophyllase.

**Table 7:** Pearson correlation matrix of pheophytinase activity and color as well as chlorophyll components in the chili pepper fruit during a 0–21 d storage period.

	PPH Activity vs. L* Value	PPH Activity vs. a* Value	PPH Activity vs. b* Value	PPH Activity vs. Chlorophyll a Content	PPH Activity vs. Chlorophyll b Content	PPH Activity vs. Total Chlorophyll Content
**Pearson r**	0.63	0.62	0.59	−0.76	−0.51	−0.80
***p*** **-value**	<0.0001	<0.0001	<0.0001	<0.0001	0.001	<0.0001

Note: PPH, Pheophytinase.

**Table 8:** Pearson correlation matrix of Mg-dechelatase activity and color as well as chlorophyll components in the chili pepper fruit during a 0–21 d storage period.

	MDcase Activity vs. L* Value	MDcase Activity vs. a* Value	MDcase Activity vs. b* Value	MDcase Activity vs. Chlorophyll a Content	MDcase Activity vs. Chlorophyll b Content	MDcase Activity vs. Total Chlorophyll Content
**Pearson r**	0.64	0.71	0.48	−0.61	−0.44	−0.65
***p*** **-value**	<0.0001	<0.0001	0.002	<0.0001	0.005	<0.0001

Note: MDcase, Mg-dechelatase.

## Data Availability

The original contributions presented in the study are included in the article, further inquiries can be directed to the corresponding author.
